# Infant Feeding*

**Published:** 1891-11

**Authors:** W. A. D. Pierce

**Affiliations:** Philadelphia


### INFANT FEEDING.*

EDITOR OF THE HOMŒOPATHIC PHYSICIAN:

I fear some one will read our dinner-table talk at Phœnixville and not only feed their infants on the richest half of the milk pure, but also drop milk altogether at the age of six or eight months and feed on broiled beef and roast lamb. Our Secretary probably put down our talk correctly, but I did not expect it to appear in print, as it has. I would suggest diluting the milk with an equal quantity of water, and that the remarks about meat were merely intended to criticise the method of feeding on corn-starch, cracker victuals, arrow-root, etc. I suggested that if starchy food was correct, the grinders would appear first; as they do not, a meaty food is more sensible.

Yours, etc.,

W. A. D. PIERCE.

PHILADELPHIA, July 30th, 1891.

* See THE HOMŒOPATHIC PHYSICIAN, August, 1891, pages 335–6.

